# Intraoperative haemodynamic monitoring and management of adults having non-cardiac surgery: Guidelines of the German Society of Anaesthesiology and Intensive Care Medicine in collaboration with the German Association of the Scientific Medical Societies

**DOI:** 10.1007/s10877-024-01132-7

**Published:** 2024-02-21

**Authors:** Bernd Saugel, Thorsten Annecke, Berthold Bein, Moritz Flick, Matthias Goepfert, Matthias Gruenewald, Marit Habicher, Bettina Jungwirth, Tilo Koch, Karim Kouz, Agnes S Meidert, Gunther Pestel, Jochen Renner, Samir G Sakka, Michael Sander, Sascha Treskatsch, Amelie Zitzmann, Daniel A Reuter

**Affiliations:** 1 Department of Anesthesiology, Center of Anesthesiology and Intensive Care Medicine, University Medical Center Hamburg-Eppendorf, Hamburg, Germany; 2 Outcomes Research Consortium, Cleveland, OH USA; 3 Department of Anesthesiology and Intensive Care Medicine, Cologne Merheim Medical Center, Hospital of the University of Witten/Herdecke, Cologne, Germany; 4 Department for Anaesthesiology, Asklepios Hospital Hamburg St. Georg, Hamburg, Germany; 5 Department of Anaesthesiology and Intensive Care Medicine, Alexianer St. Hedwigkliniken Berlin, Berlin, Germany; 6 Department of Anaesthesiology and Intensive Care Medicine, Evangelisches Amalie Sieveking Krankenhaus, Hamburg, Germany; 7 Department of Anaesthesiology, Intensive Care Medicine and Pain Medicine, University Hospital Giessen, Justus-Liebig University Giessen, Giessen, Germany; 8 Department of Anesthesiology and Intensive Care Medicine, University Hospital Ulm, Ulm, Germany; 9 Department of Anesthesiology and Intensive Care, Philipps-University Marburg, Marburg, Germany; 10 Department of Anaesthesiology, University Hospital LMU Munich, Munich, Germany; 11 Department of Anesthesiology, University Medical Center of the Johannes Gutenberg-University, Mainz, Germany; 12 Department of Anesthesiology and Intensive Care Medicine, Municipal Hospital Kiel, Kiel, Germany; 13 Department of Intensive Care Medicine, Gemeinschaftsklinikum Mittelrhein gGmbH, Academic Teaching Hospital of the Johannes Gutenberg University Mainz, Koblenz, Germany; 14 Department of Anesthesiology and Intensive Care Medicine, Charité – Universitätsmedizin Berlin, corporate member of Freie Universität Berlin, Humboldt Universität zu Berlin, Charité Campus Benjamin Franklin, Berlin, Germany; 15 Department of Anaesthesiology, Intensive Care Medicine and Pain Therapy, University Medical Centre of Rostock, Rostock, Germany

**Keywords:** Arterial pressure, Cardiac output, Echocardiography, Haemodynamic monitoring, Heart rate, Stroke volume

## Abstract

Haemodynamic monitoring and management are cornerstones of perioperative care. The goal of haemodynamic management is to maintain organ function by ensuring adequate perfusion pressure, blood flow, and oxygen delivery. We here present guidelines on “Intraoperative haemodynamic monitoring and management of adults having non-cardiac surgery” that were prepared by 18 experts on behalf of the German Society of Anaesthesiology and Intensive Care Medicine (Deutsche Gesellschaft für Anästhesiologie und lntensivmedizin; DGAI).

## Background

Haemodynamic monitoring and management are cornerstones of perioperative care. The goal of haemodynamic management is to maintain organ function by ensuring adequate perfusion pressure, blood flow, and oxygen delivery to prevent perioperative complications – that remain as high as almost 20% in patients having elective non-cardiac surgery [1, 2].

We identified clinically important questions on intraoperative haemodynamic monitoring and management of adults having non-cardiac surgery, discussed them in a group of experts, and formulated consensus recommendations based on current evidence.

## Methods

Initiated by the German Society of Anaesthesiology and Intensive Care Medicine (Deutsche Gesellschaft für Anästhesiologie und lntensivmedizin; DGAI) these guidelines were prepared between April 2022 and October 2023 by four coordinators (BS, DAR, MS, BJ) and 14 experts (all co-authors). We registered this project as S-class 1 guidelines at the Association of the Scientific Medical Societies in Germany (http://www.awmf.org). S-class 1 guidelines are expert recommendations developed by a representative group of experts based on informal consensus without formally assessing the quality of evidence.

We assumed that experts and clinicians may not necessarily consider the same topics clinically important. In May 2022, we, therefore, asked anaesthesiologists at three German university hospitals (Giessen, Hamburg, and Rostock) to submit questions on intraoperative haemodynamic monitoring and management they would expect to get answered in guidelines. The submitted questions were used to formulate 19 core questions. We discussed and answered these 19 questions (a) within groups of 2–3 experts and (b) within the group of all experts in two plenary meetings in September 2022 (virtual) and January 2023 (in-person). The revised final consensus recommendations (considering comments of the board and working groups of the DGAI) were agreed on by the experts and approved by the DGAI in September 2023. The guidelines (including a table detailing each expert’s conflicts of interest) were published online in German on October 4, 2023 (https://register.awmf.org/de/leitlinien/detail/001-049).

The strength of the recommendations is reflected by the wording – with “should”/”should not” reflecting strong recommendations, “ought”/”ought not” reflecting recommendations, and “may be considered” reflecting open recommendations [3].

## Recommendations

### How should oscillometric arterial pressure monitoring be performed?

#### Consensus recommendations

- Oscillometric arterial pressure monitoring should – if possible – be performed on the upper arm.
- For oscillometric arterial pressure monitoring, a cuff size appropriate for the circumference of the upper arm should be selected and the cuff should be placed tightly around the upper arm without contact with the olecranon.
- For oscillometric arterial pressure monitoring, the upper arm cuff should be positioned at the level of the heart and external compression or manipulation of the cuff during the measurement should be avoided.
- For patients under general anaesthesia, oscillometric arterial pressure monitoring ought to be performed every 3 min. The measurement interval should be adapted to the clinical context.

#### Summary of evidence

Automated oscillometry is the most commonly used method for non-invasive arterial pressure monitoring in perioperative medicine. Selecting a cuff adequately sized in relation to the upper arm circumference is essential for correct measurements. Using a cuff that is too small will provide erroneously high arterial pressures. Using a cuff that is too large will provide erroneously low arterial pressures [4]. The cuff should be placed around the upper arm without contact to the olecranon to allow circular compression. The cuff should be positioned at the level of the heart during the measurement. A cuff positioned above the level of the heart during measurements will provide erroneously low arterial pressure values due to hydrostatic pressure differences [5]. A cuff positioned below the level of the heart during measurements will provide erroneously high arterial pressure values [5]. External compression, vibration, or muscle activity during the measurement will cause artifacts.

In general, oscillometry systematically overestimates low arterial pressures and underestimates high arterial pressures [6–8]. This may lead to delayed detection of hypo- or hypertension.

For patients under general anaesthesia, measurements ought to be taken every 3 min. If closer arterial pressure monitoring is deemed necessary, continuous arterial pressure monitoring is indicated. The measurement interval for oscillometric arterial pressure measurement should be adapted to the clinical context.

### In which patients should arterial pressure be measured continuously?

#### Consensus recommendation

- Continuous arterial pressure monitoring should be used in all patients who – because of anaesthesiologic or surgical procedures or concomitant diseases – are at risk for complications associated with hypotension or hypertension.

#### Summary of evidence

Arterial pressure can be continuously measured invasively with an arterial catheter (reference method) or non-invasively (e.g., using a finger cuff method). Continuous arterial pressure monitoring allows detecting arterial pressure changes in real-time and can thus help reduce arterial pressure fluctuations and hypotension [9–13].

Whether continuous arterial pressure monitoring ought to be used depends on surgical and patient-specific risks for arterial pressure fluctuations [14]. Close arterial pressure monitoring is required during intracranial and vascular surgery, surgery in sitting position, and surgery with expected hypotension. Patient-specific factors for continuous arterial pressure monitoring include cardiovascular diseases, high risk for cardiovascular complications, elevated intracranial pressure (with the risk of low cerebral perfusion pressure), and aneurysms at risk of rupture. Additionally, continuous arterial pressure monitoring may also be indicated in patients in whom intermittent arterial pressure monitoring using oscillometry is inaccurate or difficult to perform (e.g., severly obese patients) [15, 16].

### How should continuous arterial pressure monitoring be performed?

#### Consensus recommendations

- Continuous arterial pressure monitoring ought to be performed with an arterial catheter.
- In low or moderate risk patients, non-invasive continuous arterial pressure monitoring may be considered.

#### Summary of evidence

Direct intraarterial arterial pressure measurement with an arterial catheter is the clinical reference method for measuring arterial pressure [17]. When used correctly, direct intraarterial arterial pressure monitoring is more accurate than non-invasive arterial pressure measurement. When correctly measured intraarterial and non-invasive arterial pressure measurements differ, intraarterial arterial pressure measurements should be used to make treatment decisions.

Non-invasive continuous arterial pressure measurements are not interchangeable with intraarterial measurements [18, 19], but may be considered for continuous arterial pressure monitoring in low or moderate risk patients.

### Should the arterial catheter for intraarterial arterial pressure monitoring be inserted before induction of anaesthesia?

#### Consensus recommendation

- In patients with an indication for intraarterial arterial pressure monitoring, the arterial catheter should be inserted before induction of anaesthesia.

#### Summary of evidence

Hypotension after induction of anaesthesia is common and associated with postoperative organ injury [20, 21]. In contrast to hypotension during surgery, hypotension after induction of anaesthesia is mainly caused by anaesthetic management and can thus be anticipated [22]. Intermittent oscillometric arterial pressure monitoring may miss hypotension – especially because it systematically overestimates low arterial pressures [6, 10, 11, 13]. In a randomised trial of 242 non-cardiac surgery patients, continuous intraarterial – compared to intermittent oscillometric – arterial pressure monitoring significantly reduced the duration and severity of hypotension during induction of anaesthesia [10].

### Which artery should be used for intraarterial arterial pressure monitoring?

#### Consensus recommendation

- Arterial catheters should primarily be inserted in the radial artery.

#### Summary of evidence

In general, arterial catheters used in perioperative and intensive care medicine are associated with a low risk for complications due to catheter insertion [23–25]. Insertion of arterial catheters into the radial artery is associated with a lower risk compared to catheter insertion in the brachial or femoral artery (2.7 per 10,000 radial, 9.0 per 10,000 femoral, and 12.3 per 10,000 brachial artery catheters) [24]. The radial artery may also be preferred because of the collateral circulation of the hand, its easy accessibility, and good possibility for compression in case of bleeding. Arterial catheters with a smaller diameter (20 G) are associated with fewer complications than arterial catheters with a larger diameter (18 G) [24]. Arterial catheters ought to be inserted using ultrasound guidance [26–28].

### What sources of error must be considered when intraarterial arterial pressure monitoring is used?

#### Consensus recommendations

- The pressure transducer should always be checked for correct levelling or zeroing.
- The dynamic response of the measurement system should be closely checked.

#### Summary of evidence

The pressure transducer must be levelled or zeroed to ensure correct intraarterial arterial pressure monitoring [29]. Levelling and zeroing procedures differ dependent on whether pressure transducers are used without or with a zero-line (i.e., a fluid-filled tube) [29].

If used without a zero-line, the pressure transducer must be positioned at the reference level (usually the right atrium [30]) to account for hydrostatic pressure differences [29]. A height difference of 10 cm between the transducer and the reference level results in an arterial pressure difference of approximately 7.5 mmHg.

If the pressure transducer is used with a zero-line, the zero-line is attached to the patient with its free end at the reference level and the zeroing function on the monitor is activated. Using a zero-line can be advantageous for patients in whom the pressure transducer cannot be positioned accurately during surgery [31]. Whenever the patient is being moved relative to the height of the pressure transducer, a new zeroing manoeuvre must be performed.

The measurement system should be closely checked for an adequate dynamic response – e.g., by means of a fast-flush test – to avoid damping phenomena [29, 32]. An underdamped system may overestimate systolic arterial pressure and underestimate diastolic arterial pressure [29]. Common reasons for underdamping include stiff tubing or modifications of the measurement system by adding additional tubes and stopcocks [29]. An overdamped system may underestimate systolic arterial pressure and overestimate diastolic arterial pressure [29]. Common reasons for overdamping include low infusion bag pressure, air bubbles in the measurement system, blood clots, lose or open connections, and kinking or obstruction of the catheter [29].

### How must the position of the patient be considered when measuring arterial pressure?

#### Consensus recommendations

- Especially during changes in patient position, arterial pressure ought to be measured closely or – even better – continuously.
- In all positions where the usual reference level “right atrium” is lower than the cranial base, non-invasively measured mean arterial pressure should be corrected for the difference in hydrostatic pressure or the reference level of the continuous arterial pressure measurement should be set at the level of the cranial base.

#### Summary of evidence

Various intraoperative positions (e.g., sitting position, prone position, lateral position, and corresponding modifications) can contribute to haemodynamic instability [33–35]. In positions with elevated upper body, a major cause of hypotension is the redistribution of blood from central to peripheral compartments with a consecutive decrease in cardiac preload. In flank and prone positions, compression of the inferior vena cava may partially obstruct venous return. Not only positioning itself but also repositioning can lead to a decrease in arterial pressure. Therefore, arterial pressure ought to be monitored closely or even continuously during changes in patient position [36].

Pressure differences between central and cerebral mean arterial pressure occur in all positions in which the head is higher than the usual reference level “right atrium” due to hydrostatic pressure differences. In these positions, mean arterial pressure targets should be corrected for hydrostatic pressure differences or the reference level should be set at the level of the cranial base to correctly monitor arterial pressure in the circle of Willis [37, 38]. Either procedure ought to be documented in the anaesthesia record. In contrast, with head-down positions, arterial pressure targets should not be corrected, and the right atrium should be used as the reference level [39].

### Which arterial pressure component should be used for arterial pressure management?

#### Consensus recommendation

- Mean arterial pressure should be used for intraoperative arterial pressure management.

#### Summary of evidence

A retrospective database study showed that the strength of the association between hypotension and acute kidney or myocardial injury is similarly strong when hypotension is defined using mean or systolic arterial pressure – but substantially weaker when using diastolic arterial pressure [40].

Intraoperative arterial pressure management should be guided by mean arterial pressure, as mean arterial pressure is the inflow pressure for most organ systems. In addition, measurement of mean arterial pressure is less dependent on the measurement location than measurement of systolic and diastolic arterial pressure as mean arterial pressure changes only slightly along the arterial tree – while systolic arterial pressure increases progressively towards the periphery and diastolic arterial pressure decreases [41].

### Which arterial pressure value should be targeted?

#### Consensus recommendation

- Mean arterial pressure should be maintained above 65 mmHg.

#### Summary of evidence

Observational research provides compelling evidence that intraoperative hypotension is associated with organ injury [21, 40, 42–51]. The association between hypotension and organ injury depends on the severity and duration of hypotension [21, 40, 42–51]. On a population level, harm thresholds for acute kidney injury and acute myocardial injury are 60–70 mmHg for mean arterial pressure and 90–100 mmHg for systolic arterial pressure [40]. Harm thresholds for individual patients remain unknown. There are only few randomised trials on targeted intraoperative arterial pressure management [52–54].

In a trial of 458 high-risk patients, keeping intraoperative mean arterial pressure ≥ 75 mmHg – compared to ≥ 60 mmHg – did not reduce the incidence of a composite outcome of acute myocardial injury, acute kidney injury, other cardiovascular complications, or death [52]. The POISE-3 trial [54] found no clinically important difference in the incidence of severe cardiovascular complications between patients randomised to a hypotension avoidance strategy with an intraoperative mean arterial pressure target of ≥ 80 mmHg and a hypertension avoidance strategy with a mean arterial pressure target of ≥ 60 mmHg.

As preoperative arterial pressure substantially varies among individuals [20], individualised arterial pressure management may reduce hypotension-associated complications. In a multicentre trial of 198 non-cardiac surgery patients, individualised arterial pressure management (based on a single preoperative systolic arterial pressure value) reduced the incidence of a composite outcome of systemic inflammatory response syndrome and organ dysfunction within one week after surgery compared to routine arterial pressure management [53].

Considering the association between intraoperative hypotension and organ injury, clinicians should maintain mean arterial pressure above 65 mmHg or systolic arterial pressure above 90–100 mmHg [17, 55].

There is no clear evidence that intraoperative hypertension is associated with organ injury in non-cardiac surgery patients [56]. A general upper intervention threshold for arterial pressure cannot be recommended.

### Which heart rate value should be targeted?

#### Consensus recommendations

- Bradycardia should be treated when it is accompanied by clinically important hypotension, reduced perfusion, or reduced oxygen delivery.
- If tachycardia is present, hypovolaemia should be excluded.

#### Summary of evidence

Heart rate, together with stroke volume, is a primary determinant of cardiac output and thus oxygen delivery [57]. In the general adult population, bradycardia is usually defined as a heart rate < 60 beats per minute [58] and tachycardia as a heart rate > 100 beats per minute [59]. These thresholds are usually also considered during surgery. There is insufficient evidence to recommend heart rate target values.

Perioperative bradycardia is common [60] and often caused by general anaesthesia [61–63], vasoactive drugs [64], and surgery [65]. Bradycardia can lead to hypotension [66] and a critical decrease in oxygen delivery if the heart rate-related reduction in cardiac output cannot be compensated by an increase in stroke volume [67].

Perioperative tachycardia may be caused by hypovolaemia, inadequate depth of anaesthesia or insufficient analgesia, or a systemic inflammatory response. Critical shortening of diastole may result in insufficient myocardial oxygen delivery with concomitant increased oxygen consumption which should be considered especially in patients with diastolic dysfunction or heart failure [67–69].

### In which patients should stroke volume/cardiac output be monitored?

#### Consensus recommendation

- Stroke volume/cardiac output monitoring may be considered in patients with a high risk for complications.

#### Summary of evidence

The decision to monitor stroke volume/cardiac output ought to be based on the risk for intra- and postoperative complications [70]. The risk for complications depends on patient-related and surgery-related risk factors. Patient-related risk factors include the underlying diagnosis requiring surgery, age, and cardiovascular or pulmonary comorbidities [70–72]. Surgery-related risk factors include invasiveness of the procedure, risk for high blood loss, or emergency surgery [71, 73].

Stroke volume/cardiac output monitoring may be considered when the individual patient-related or surgery-related risk is high. Especially in patients with a high risk for perioperative complications, stroke volume/cardiac output-guided haemodynamic management may reduce the risk for postoperative complications [74, 75]. Monitoring stroke volume/cardiac output alone will not improve postoperative outcomes [76].

### Which stroke volume/cardiac output value should be targeted?

#### Consensus recommendations

- Stroke volume/cardiac output target values should be individually defined for each patient.
- Stroke volume/cardiac output should be interpreted in the context of clinical and metabolic signs of hypoperfusion.
- Stroke volume/cardiac output should not be routinely maximised.

#### Summary of evidence

Stroke volume/cardiac output is regulated to meet metabolic needs. Resting cardiac output depends on age, sex, and comorbidities [77, 78]. Haemodynamic therapy guided by stroke volume/cardiac output thus ought to consider variables of oxygen delivery and oxygen consumption. The targeted stroke volume/cardiac output value ought to be high enough to ensure sufficient oxygen delivery for the individual patient in each situation.

Stroke volume/cardiac output-guided haemodynamic management may reduce postoperative complications [74, 79, 80]. Most blood flow-guided haemodynamic management concepts target maximal stroke volume/cardiac output values for the individual patient [80]. Small randomised trials suggest that cardiac output-guided haemodynamic management targeting preoperative baseline or post-induction cardiac index may reduce postoperative complications compared to routine care [81, 82]. Optimal stroke volume/cardiac output target values remain unclear, and may depend on the degree of surgical trauma and systemic inflammation [83]. We recommend not routinely targeting individual maximal values (OPTIMISE II [84] – results presented at the EBPOM World Congress of Prehabilitation Medicine 2023 in London on July 6, 2023) or „supranormal“ values [85–87].

### Which tests should be used to assess fluid responsiveness?

#### Consensus recommendations

- To assess fluid responsiveness, dynamic preload variables (e.g., pulse pressure variation or stroke volume variation) should be used in mechanically ventilated patients.
- If dynamic preload variables cannot be used, stroke volume or cardiac output-based fluid challenge tests ought to be performed to assess fluid responsiveness.
- Static preload variables (e.g., central venous pressure) should not be used to assess fluid responsiveness.
- Even in fluid-responsive patients, the indication for fluid administration should be determined individually based on haemodynamics and clinical context.

#### Summary of evidence

The dynamic preload variables pulse pressure variation (PPV) and stroke volume variation (SVV) can be used to predict fluid responsiveness in mechanically ventilated patients with tidal volumes of at least 8 ml/kg and sinus rhythm [88–91]. Thresholds to distinguish between fluid responsive and non-responsive patients are 11% (8–15%) for PPV and 11% (7.5–15.5%) for SVV [92]. However, there are grey zones around these thresholds in which the ability of PPV and SVV to predict fluid responsiveness is limited [93]. Limitations for the use of PPV and SVV – such as low heart rate/respiratory rate ratio, irregular heart rhythm, mechanical ventilation with low tidal volumes, increased abdominal pressure, open chest conditions, and spontaneous breathing – need to be considered in clinical practice [94, 95].

If dynamic preload variables cannot be used, a fluid challenge considering fluid-induced changes in stroke volume/cardiac output ought to be performed to assess fluid responsiveness [96–98]. During a fluid challenge, a certain amount of fluid is administered over a short time and changes in stroke volume/cardiac output are evaluated [97, 98]. An increase in stroke volume of 10–15% is considered a sign of fluid responsiveness. However, fluid challenges are not only a test but a therapeutic intervention bearing the risk to give fluids to patients who are not fluid responsive [99, 100]. In some patients, an increase in stroke volume may cause a (physiological) reduction in heart rate resulting in no net effect on cardiac output. It is thus useful to evaluate both stroke volume and cardiac output when assessing fluid responsiveness.

Static pressure-based and volumetric preload variables, such as central venous pressure or global end-diastolic volume, should not be used to assess fluid responsiveness, but still are important in the assessment of haemodynamics [89].

Fluid administration should always be indicated carefully – also in patients with signs of fluid responsiveness – considering other haemodynamic variables and the clinical context; fluid responsiveness does not equal the need for fluids.

### When should intraoperative echocardiography be performed?

#### Consensus recommendations

- Echocardiography should be performed in patients with haemodynamic instability not responding to initial treatment, especially when the cause of haemodynamic instability is unclear.
- Echocardiography may be considered to guide haemodynamic therapy.
- Echocardiography images and results should be saved in the patient’s medical records.

#### Summary of evidence

During surgery, transthoracic echocardiography (TTE) or transoesophageal echocardiography (TEE) can be used to directly assess cardiac anatomy and function [101]. TTE is non-invasive and therefore ought to be preferred [102, 103].

Intraoperative focused echocardiography should be performed in patients with haemodynamic instability not responding to initial treatment, especially when the cause of haemodynamic instability is unclear. Echocardiography may be considered to guide haemodynamic therapy.

Preoperative focused echocardiography ought to be performed in (emergency) patients with suspected or known clinically relevant cardiovascular disease to better interpret intraoperative findings during haemodynamic instability [104–109].

Echocardiography, especially TEE, should be performed by experienced examiners [110]. Training may be certified by national [111, 112] or international certificates.

### Should urine output be used to assess haemodynamics?

#### Consensus recommendation

- Urine output alone should not be used to diagnose hypovolaemia or to guide haemodynamic management.

#### Summary of evidence

Normal urine output is often considered a sign of adequate volume status and cardiac output. Oliguria may result from haemodynamic instability with reduced renal perfusion. In addition, increased intra-abdominal pressure, e.g., during laparoscopic procedures, can cause transient oliguria. During surgery, possible causes of oliguria should be considered and treated if necessary. Intraoperative oliguria is associated with postoperative acute kidney injury in patients having non-cardiac surgery [113, 114]. However, intraoperative oliguria is a poor predictor of postoperative acute kidney injury [115]. Targeting urine output does not reduce acute kidney injury [116, 117] or mortality [118].

### Should lactate be used to assess haemodynamics?

#### Consensus recommendations

- When hypoperfusion or inadequate tissue oxygenation are suspected, lactate should be measured to assess haemodynamics.
- Elevated lactate should be interpreted considering possible non-haemodynamic causes.

#### Summary of evidence

Lactate levels > 2 mmol/l are considered pathological. Perioperative lactataemia may indicate anaerobic metabolism as a result of hypoperfusion, insufficient oxygen delivery or impaired oxygen utilisation [119, 120]. Perioperative lactataemia, therefore, ought to be evaluated as a sign of hypoperfusion and possible risk factor for postoperative complications – considering other diagnoses such as liver failure, medications, or thiamine deficiency [121, 122]. In addition to single lactate measurements, lactate clearance is an important prognostic factor. During haemodynamic instability or lactataemia, lactate measurements ought to be repeated in a regular interval, e.g., at least every two hours as recommended in patients with shock [123]. Elevated lactate is an unspecific marker that always ought to be interpreted in context with haemodynamic variables.

### Should central venous oxygen saturation be used to assess haemodynamics?

#### Consensus recommendation

- If hypoperfusion or inadequate tissue oxygenation is suspected, central venous oxygen saturation may be used for additional assessment of haemodynamics.

#### Summary of evidence

Central venous oxygen saturation (S_cv_O_2_) reflects the global ratio of oxygen delivery and oxygen consumption [124]. There is thus a close relationship between S_cv_O_2_ and cardiac output. At the same time, S_cv_O_2_ also reflects oxygen uptake and metabolism [125]. Normal S_cv_O_2_ (> 70%) does not exclude regional hypoperfusion or tissue hypoxemia [126]. S_cv_O_2_ is also influenced by other factors, such as arterial oxygen content, body temperature, and level of sedation [124, 127]. Low S_cv_O_2_ is associated with complications in patients having non-cardiac surgery [128, 129]. However, in a trial of 241 patients, S_cv_O_2_-guided fluid therapy did not reduce postoperative complications compared to routine care in patients having colorectal surgery [130].

### What is the role of monitoring the microcirculation using vital microscopy?

#### Consensus recommendation

- Monitoring the microcirculation using vital microscopy should not be used to guide haemodynamic therapy.

#### Summary of evidence

The functionality of the microcirculation is essential for a sufficient oxygen and nutrition exchange and thus organ function [131]. The regional microcirculation can be visualised and quantified using vital microscopy. Direct monitoring of the microcirculation, however, is technically challenging and analysis is time-consuming [132]. Vital microscopy is usually performed in the sublingual area because it is easily accessible [132]. The sublingual microcirculation not necessarily reflects the microcirculation in other regions of the body [133, 134]. In patients having elective non-cardiac surgery, the microcirculation usually remains intact and preserved [135–138]. Further studies are needed to investigate whether therapeutic interventions targeting the microcirculation may have positive effects [139].

### Should near-infrared spectroscopy be used to assess haemodynamics?

#### Consensus recommendation

- Near-infrared spectroscopy may be considered to complement the hemodynamic assessment.

#### Summary of evidence

Near-infrared spectroscopy (NIRS) allows measurement of the regional oxygen saturation in vessels as a surrogate for tissue oxygenation. In contrast to pulse oximetry, NIRS cannot differentiate between arterial and venous oxygen saturation. Absolute values of regional oxygen saturation show a high inter-individual variability. Thus, relative changes compared to baseline values are often considered [140–142]. NIRS is most commonly used to monitor cerebral oxygen saturation. The effect of using perioperative NIRS monitoring on postoperative cerebral outcomes in adults remains to be elucidated [143]. In addition, NIRS harm thresholds as well as effects of therapeutic interventions on NIRS need to be investigated.

## Strengths and limitations

The core questions discussed in these guidelines were proposed by clinicians at three German university hospitals and thus represent a broad spectrum of anaesthesiologists with various levels of experience. The practical consensus recommendations thus focus on thematic areas which remained unclear for clinicians.

We carefully selected a representative group of 18 members of the DGAI who have a solid scientific track record in the field of intraoperative haemodynamic monitoring and management. Nevertheless, the consensus process is based on expert interpretation during discussions between a limited number of experts. There thus remains some risk of bias. Furthermore, the consensus recommendations result from unsystematic literature review with expert interpretation. We did not perform a systematic review or meta-analysis. Additionally, the consensus recommendations are based on informal consensus without formally assessing the quality of the evidence.

## Summary

Haemodynamic monitoring and management are cornerstones of perioperative care. These guidelines on “Intraoperative haemodynamic monitoring and management of adults having non-cardiac surgery” were prepared by 18 experts on behalf of the DGAI and provide expert consensus recommendations addressing 19 core questions around intraoperative haemodynamic monitoring and management.

## Data Availability

No datasets were generated or analysed.
